# Theories of truth and teaching clinical reasoning and problem solving

**DOI:** 10.1007/s10459-018-09871-4

**Published:** 2019-01-22

**Authors:** Eugène J. F. M. Custers

**Affiliations:** Centre for Research and Development of Education, University Medical Centre Utrecht, PO Box # 85500, 3508 GA Utrecht, The Netherlands

**Keywords:** Clinical reasoning, Medical problem solving, Diagnostic competence

## Abstract

In this paper, we will first discuss two current meta-theories dealing with different, aspects of “truth”. The first metatheory conceives of truth in terms of coherence (rationality, consistency): a body of knowledge is true when it contains no inconsistencies and has at least some credibility. The second metatheory conceives of truth as correspondence, i.e., empirical accuracy. The two metatheories supplement each other, but are also incommensurable, i.e., they cannot be expressed in each other’s terms, for they employ completely different criteria to establish truth (Englebretsen in Bare facts and naked truths: a new correspondence theory of truth, Routledge, London, 2005). We will discuss both the role of both metatheories in medicine, in particular in medical education in a clinical context. In line with Hammond’s view (Med Decis Mak 16(3):281–287, 1996a; Human judgment and social policy: irreducible uncertainty, inevitable error, unavoidable injustice, Oxford University Press, New York, 1996b), we will extend the two metatheories to two forms of *competence*: coherence competence and correspondence competence, and demonstrate that distinguishing these two forms of competence increases our insights as to the best way to teach undergraduate students clinical problem solving.

If we hold a belief about a patient’s diagnosis, make a judgment of a patient’s state, or decide on a treatment, how do we know that this belief, judgment, or decision is “true” or “correct”? Or, even more fundamental: What do we mean by “true” when we say, “the true diagnosis was…”? Some philosophers, most notably post-structuralists, will claim there is no such thing as a “true diagnosis.” However, we believe this view to be rather unconstructive and will adopt a more pragmatic stance, i.e., that diseases are entities that exist in the empirical word (“out there”) and that we all—doctors, students, patients, teachers, anyone—can obtain at least *indirect* knowledge of the presence of a disease through observations—of signs, symptoms, patient behaviors, laboratory values, contextual aspects. Apart from indirect, this knowledge is also *fallible*, and hence our diagnoses are always more or less uncertain: the world in which we live is characterized by “irreducible uncertainty” (Hammond 1996b). Rather than speculating whether it makes sense to say that a diagnosis is “true,” it seems more fruitful to focus on the *criteria* we use to judge whether a diagnosis (or any other medical decision) is true in the sense: correct. The consequences of this approach are twofold: first, it enables us to hold different versions of truth, as a consequence of using different criteria, while refraining from philosophical speculation. Second, it enables us to develop a new view of judging diagnostic *competence*, and we will try to demonstrate that if we can judge diagnostic competence, we can also improve it through education or experience.

## Correspondence and coherence theories of truth

In line with contemporary philosophy of science, we distinguish two major frameworks that differ in the criteria they use to decide what is true: the *correspondence* theory of truth (Prior 1967) and the *coherence* theory of truth (Rescher 2002; White 1967). In fact, these are metatheories or frameworks, rather than theories: they are not themselves testable theories, but can be used to derive substantive theories that can be tested. The correspondence and coherence metatheories use entirely different criteria to judge the truth of a particular theory and the hypotheses, statements, or claims derived from this theory. Theories are always tested and contrasted within their respective frameworks; there is no common standard that can be used to decide whether any theory within the correspondence metatheory is more “true” than any theory within the coherence framework. In other words, both metatheories are incommensurable (cf. Kuhn 1962, pp. 148–150).

Theories within the correspondence framework use *empirical accuracy* as the ultimate standard for judging truth (e.g., Hammond 1996a, b; Mosier 2009; Vicente 1990). For example, How high is this patient’s blood sugar? Was the blood culture positive for *Staphylococci*? What is the accurate diagnosis given these symptoms? Did the clinician get it right? To arrive at valid judgments about the natural world, we use cues in the environment that say something about this world. The assumption is that the natural world (including patients in medical practice, the “natural world” for the clinician) offers tangible indicators of its intangible aspects, such as diseases (Hammond 1996a). For example, if I claim, “this patient suffers from an infection with the West Nile virus,” I will point toward these cues, e.g., high fever, headache, a rash, meningitis, the patient having been in a region where mosquitoes are ubiquitous or even remembering a mosquito-bite, and, ultimately, the results of serologic testing. None of these cues uniquely determines diagnostic accuracy, and even their joint presence leaves room for uncertainty (though the serologic test, if properly performed and reported, may reduce this to close to zero). Most importantly, working within the correspondence metatheory, I do not need to explain *how* the cues hang together, or *how* I arrived at this diagnosis, and maybe I am even not able to do so at all; the only thing that counts whether it is empirically accurate or not, given a particular standard against which this accuracy can be assessed (Hammond 1996a). Though within the correspondence framework little can be said about the inner workings of the diagnostic process, researchers are able to empirically model and predict its outcomes, e.g., by putting the cues into a regression equation and determining the fit between the predicted values and the actual outcomes (e.g., Dawes et al. 1989; Peterson et al. 1965). By calculating the weights that each cue should be assigned, the correspondence approach affords to draw inferences about the origins of inaccuracies and why physicians disagree—without investigating their reasoning or aiming at consensus (Kirwan et al. 1988). It is even possible to discover how students learn and to improve their accuracy by providing feedback (Tape et al. 1992, 1995), again without asking for any justification (Wigton 1988, 1996).

Theories within the coherence framework use *rationality* or *logical consistency* as the ultimate standard for judging truth. As this is a fairly weak criterion, it is in practice often extended to coherence, a stronger criterion. That is, two statements can be logically consistent, while still lacking coherence. For example, taken together, “Mary has an excellent appetite,” and “Mary has lost weight recently,” may lack coherence, but they can be made coherent by adding new, explanatory information (e.g., “Mary has been diagnosed with hyperthyroidism”). Full inconsistencies cannot be solved this way; if two claims are inconsistent, they contradict each other, and hence at least one must be false (e.g., “Mary has been diagnosed with *hyper*thyroidism” and “Mary has been diagnosed with *hypo*thyroidism”). Unfortunately, coherence is much harder to define than consistency (for attempts, see Meijs 2006; Thagard and Verbeurgt 1998), and we will treat it somewhat intuitively here, its meaning being similar to “credibility” (Evans et al. 1983). A theory, or line of reasoning, is coherent when it is credible. For this reason, theories within the coherence framework often start from the mind of the decision maker; in medicine the mind of the clinician. Many studies on medical problem solving (e.g., Elstein et al. 1978) and early AI programs, such as MYCIN (Shortliffe and Buchanan 1975) and INTERNIST (Miller et al. 1982), have tried to model diagnostic reasoning processes to achieve coherence. In medical diagnosis, coherence approaches are not necessarily deterministic; uncertainty can be modelled by using Bayes’ theorem as the normative standard of rationality (Hammond 1996a; Kahneman and Tversky 1972).

## Correspondence and coherence competence

At first sight, this epistemological discussion does have few ramifications for medical education. However, in 1996, Kenneth Hammond made a major move by directly connecting the two metatheories to human expertise (Hammond 1996a). In his view, coherence and correspondence are not just criteria for judging the validity of claims, they also refer to two different types of *competence*. In short, correspondence *competence* is an individual’s ability to accurately judge and respond to cues in the environment, and the empirical accuracy of these judgments is the standard by which correspondence competence is evaluated (Mosier 2009, p. 154). Coherence *competence*, on the other hand, refers to the ability to come up with a line of reasoning that is free of inconsistencies (a necessary, but not sufficient, requirement for reasoning to be coherent), defensible (e.g., the reasoning is anchored in canonical knowledge accepted by a professional community, and the reasoner can make clear how the separate steps in the reasoning are connected), and leads to credible conclusions even in the absence of knowledge about their actual empirical accuracy (Mosier 2009, pp. 154–155). For example, ultimately there is no way to decide on the empirical accuracy of a diagnosis of a simulated or fictitious patient—the ‘truth’ of this diagnosis can only be judged from a coherence perspective. If, in an educational context, feedback is used as a means to improve coherence competence, it is always in the form of process feedback. Within the medical profession (and probably within most other professions as well), experts will agree upon the criteria that define what is correct and what is incorrect diagnostic reasoning even if they arrive at different diagnoses for the same case (cf. Kanter et al. 2010).

Diagnostic accuracy that results from correspondence competence is often dubbed “clinical acumen” and it typically defies explanation in coherence terms. Expert clinicians are able to “size up” patients or, to put it more formally, activate appropriate illness scripts early in the diagnostic encounter on basis of only a few early available cues, mostly from the context of the patient (Hobus et al. 1987; Custers 2015). This form of competence is often attributed to practitioners’ using implicit or tacit knowledge (Berry 1987; Engel 2008; Goldman 1990; Polanyi 1969). Similarly, diagosticians are assumed to use common heuristics, such as how easily a certain disease comes to mind (the availability heuristic; Tversky and Kahneman 1973) or how representative a patient is of a particular disease (the representativeness heuristic; Kahneman and Tversky 1972). These heuristics are applied unconsciously and it is very hard to predict when they will work (i.e., lead to accurate diagnoses) and when not, though it is clear they are often successful (Gigerenzer et al. 1999). The distinction between coherence and correspondence competence explains why post hoc descriptions of the diagnostic process are notoriously unreliable: for example, asking a clinician to explain how he arrived at a particular diagnosis when he relied on correspondence competence may reveal that he is “right for the wrong reason” (Hammond 2007, pp. 40–43), or that it is “not necessarily a bad decision, but one poorly made” (Dijksterhuis 2004, p. 586). It also explains why violating a specific coherence standard (e.g., Bayesian reasoning) may be irrelevant when diagnostic accuracy is the only criterion against which the clinician’s performance is judged. This also implies that correspondence competence can never be assessed on basis of a student’s or expert’s performance on a single case; rather, a large—representative—sample of cases is necessary, in particular if the probability of arriving at the correct solution by informed guessing is relatively high. Inaccuracies or errors in the correspondence sense are usually a consequence of degraded task conditions: lack of information, unreliability of the indicators, ambiguous observations; or, in general: uncertainty in the world out there (Hammond 1996a). For example, Sanders (2009, pp. 69–74) describes a case where the attending resident couldn’t properly examine a patient because this patient couldn’t stand the light—and hence, he missed some classic symptoms, which prevented him from exerting his full correspondence competence. It didn’t affect the resident’s coherence competence, though: that he missed the diagnosis was not because of flawed reasoning or lack of knowledge. This is why coherence theorists have troubles dealing with such errors (cf. the “no-fault errors” identified by Graber et al. 2005), whereas within the correspondence framework these errors “just occur” as a consequence of the irreducible uncertainty in our ecology (Schiff et al. 2005, p. 263; Hammond 1996b).

When coherence competence is required, the student or medical expert must demonstrate that he or she knows how a system—i.e., the human body—works, and must be able to describe the functional relations among the variables in that system. Thus, for example, when a medical student describes a patient’s symptoms to his or her teacher, the teacher may query by asking, “do you know the mechanism for that,” thus testing the coherence of the student’s domain knowledge (Hammond 2000, p. 33). Many expertise studies contrast expert and novice clinicians’ performance on a specific form of coherence competence, e.g., the role of biomedical knowledge in clinical reasoning (e.g., Kaufman and Patel 1991; Patel et al. 1997; Woods et al. 2007a, b), or investigate whether physicians can correctly apply Bayesian principles (Eddy 1982; Hammond 1996b). Coherence errors are conceived as process failures or even as violations of rationality: failure to acknowledge the relevance of certain information, failure to apply knowledge, and flawed reasoning, often construed as biases (e.g., Christensen et al. 1991; Cutrer et al. 2013; Johnson et al. 1992; Graber et al. 2002; Redelmeier 2005). As achieving coherence requires much effort and is intrinsically fragile (i.e., a particular line of reasoning put forth by an expert may easily be challenged by another expert), researchers who work within this framework are generally more interested in errors and more pessimistic about practitioners’ competence than those who work within the correspondence framework. This also explains why the studies by Kahneman and Tversky—who investigated human biases and irrationality and hence worked within the coherence metatheory (Hammond 1996b), have become so popular in medical education. To avoid making errors in medical diagnosis and decision making, health practitioners often rely on protocols, that is, on others who have already performed the hard coherence work for them. In this sense, holding on to protocols is not “cookbook medicine” but exploiting the community’s coherence competence. Researchers who investigate practitioners’ competence from a correspondence point of view, on the other hand, are more inclined to believe that practitioners are generally competent (e.g., Croskerry 2002; Mamede et al. 2010; Hammond 1996a, p. 282; McGuire 1985), whether or not they attend to protocols.

It may be tempting to identify correspondence competence with “intuition” or “System 1 thinking” and coherence competence with “analysis” or “System 2” thinking (Eva 2005; Kahneman 2011; Norman 2009), but this is not the case. The criterion to assess correspondence competence is empirical accuracy, irrespective of how this is achieved: through intuition or through analysis. A good example of the latter is the work on clinical versus actuarial prediction (Dawes et al. 1989; see also Goldberg 1970): Statistical models can outperform clinicians in making accurate predictions. Regression analyses are regularly used in several domains to achieve correspondence competence; similarly, “big data” is used to discover patterns in large arrays of data not easily caught by humans using their intuition. Conversely, if human beings have to repeat the same analysis many times, they will develop intuitions that represent coherence competence: they will be able to jump to a conclusion without the need to redo the full analysis time and again, which is why Simon (1987, p. 63) has dubbed this form of intuition “analysis frozen into habit.” Of course, this is not so say that *all* human intuitions are frozen analyses; there are also “natural” intuitions developed by experience or even ingrained in human nature (for a discussion of different types of intuition and associated measurement instruments, see Pretz et al. 2014).

## Correspondence and coherence competence: Do the two meet?

In general, the assumption appears to be that good (i.e., coherent) clinical reasoning will inevitably result in correct (i.e., accurate) diagnosis. For example, according to McGuire (1985), “(…) there should be a close and predictable connection between diagnostic accuracy and the quality of the data collection and management employed in reaching that diagnostic outcome.” Quality of the management is a typical feature of coherence competence, and will be judged against a coherence standard. However, even in physics, which is featured by a generally strong relationship between coherence (mathematical theory) and correspondence (experimental results), rigorous reasoning and experimental outcomes do not necessarily align. This is exactly the reason why the Nobel Committee demands experimental verification and does not award the Nobel Prize to research that is outside the scope of experimental testing, no matter how exceptional the candidate recipient’s coherence competence might be. In more mundane domains, such as medicine, the connection between coherence competence and correspondence competence is definitely more tenuous than in physics. Quite a few questionable practices in medicine, for example, are easy to defend from a coherence point of view, yet are not supported by empirical evidence (Tape 2009). In medical diagnosis, simple heuristics, that leave aside much of a practitioner’s coherence competence, often lead to more accurate decisions and judgments than more resource-intensive—i.e., more coherent—processing strategies (Gigerenzer and Brighton 2009). Similarly, Norman et al. (1989) found a negative relationship in expert dermatologists between response time and diagnostic accuracy, revealing the superiority of correspondence competence of practitioners in this domain (quick and efficient, with only a small risk of failure; Lopes 1991) in contrast to their coherence competence (time consuming, effortful, and resulting in less accurate outcomes). In short, though in medicine both forms of competence *can* converge on the same diagnosis, this should not be taken as a rule, and coherence and correspondence competence often will have to be assessed independently (e.g., Christensen-Szalanski 1986; Shaffer and Hulsey 2009). Most notably, even experts can harbor serious misconceptions at a deep knowledge level, apparently without compromising their correspondence competence (Feltovich et al. 1989; Spiro et al. 1989; Patel and Kaufman 1995).

## Implications for medical education

To begin with, and maybe most importantly, clinical teachers should be aware that medicine is an example of a hybrid ecology (Mosier 2009, p. 161); in such an ecology, coherence can be viewed as a strategy to support correspondence, to reduce the uncertainty inherent in the correspondence world, while never becoming a full substitute for it. Extended training and technical innovations all increase the likelihood of alignment between a clinician’s coherence and correspondence judgments, but ultimately both are indispensible for clinical competence. The clinical teacher should be aware of the difference between the two forms of competence, and in particular of the different standards against which each is judged, and that these standards are—in education as well as in practical clinical work—complementary. In addition, both forms of competence need different training formats for optimal development.

Coherence diagnostic competence can best be trained in small-group sessions, under little or no time pressure, using paper cases (or simulations), with an emphasis on constructing a differential diagnosis, and providing process feedback: do the students identify the important information in the case, do they appropriately use their knowledge, can they defend and elaborate on the structure of their differential diagnoses? Custers et al. (2000) describe a clinical teaching approach that aims at training coherence competence and can be applied at any level of expertise. Teachers should also realize that when using written or computerized cases, much of the necessary patient information is already “pre-interpreted” to enable learners to circumvent using their correspondence competence. For example, students do not need to rely on their perceptual senses to discover signs and symptoms. Teaching in the coherence framework may involve, for example, explaining the diagnostic implications of a specific heart murmur, but it cannot be used to train students to *hear* this murmur in an actual patient. Teachers should always be aware they are training students to deal with descriptions of patients, rather than with real patients. Though teachers may test students’ coherence competence by asking hypothetical questions, e.g., “What would your primary diagnosis be if the test results were negative?,” it is not common, in this format, that students are encouraged to challenge the findings in a case per se. Finally, to foster analytical clinical reasoning, it is important the supervisor is not informed about the actual diagnosis of the patient—ideally, there is no “actual” diagnosis, for the case is “unauthentic”, i.e., constructed, or at least adapted; for students, this may be disappointing: they may want to know whether they “got it right” (in the correspondence sense). However, the risk of using an actual case with a known diagnosis is that clinical reasoning becomes a reconstruction of a remembered process, rather than unbiased diagnostic reasoning on basis of the givens in the case.

Clinical competence in the correspondence sense, on the other hand, can ultimately be learned only in contact with real, “authentic” patients, for they only provide the (uninterpreted) cues necessary to develop clinical acumen. The gold standard for an accurate diagnosis is usually the pathologist’s judgment or the outcome of one or more laboratory tests. Unlike coherence competence, correspondence competence does not assume that the clinician is consciously aware of *how* it works: in the clinic, it often requires an “intuitive jump” from the findings to the diagnosis. A large intuitive jump is subjectively experienced as a hunch (the diagnosis is suspected, but not obvious), a small intuitive jump as plain pattern recognition. The smaller the jump, the lesser the diagnostician will be inclined to question the outcome. In case of pattern recognition, there is no diagnostic process that can be retraced and investigated in retrospect for possible errors; there is only an outcome. Correspondence outcomes may be improved by the use of electronic diagnostic devices, but their role in developing correspondence competence in students is not clear, in particular if their workings are opaque and their outcomes not infallible. For learning to occur, hunches and recognized patterns need to be confirmed or disconfirmed by immediate outcome feedback; if this feedback is delayed or absent, an incorrect association between the features and the response may be inadvertently reinforced. Thus, it will be good practice to prevent inexperienced students from jumping to conclusions, and early training of this form of competence will aim at encouraging students to describe as accurately as possible what they observe (see, hear, smell, feel, etc.), rather than asking to come up with a diagnosis (or diagnostic suggestion) as quickly as possible. This is also important because studies have shown that after being informed about the correct diagnosis, students may ‘recognize’ symptoms or other features typical for this diagnosis they did not notice in advance (Brooks et al. 2000; LeBlanc et al. 2002). This may falsely suggest the learner has correspondence competence; in fact, he uses coherence competence (knowledge about the diagnosis) to infer what he was supposed to see, but in fact missed. Obviously, “real” correspondence competence requires you perceive a feature, sign, or symptom before you can activate a diagnostic hypothesis. Making the student aware of the missed feature after the fact may be helpful in developing his or her correspondence competence, but it makes no sense to ask: “Why didn’t you *see* it?” for this asks for a coherence explanation in a correspondence context. Conversely, in a coherence context, a teacher should not ask the student to come up with *the* accurate diagnosis, even if the student insists on being informed about this and the teacher claims it to be an “authentic patient;” rather, the clinician should ask the student for a likely diagnosis and—even more importantly—to defend this. Increasing teachers’ awareness about the two forms of competence will not revolutionise clinical education, but it may contribute to fine-tuning of clinical training and fostering the development of both forms of competence in students.

## References

[CR1] Berry DC (1987). The problem of implicit knowledge. Expert Systems.

[CR2] Brooks LR, LeBlanc VR, Norman GR (2000). On the difficulty of noticing obvious features in patient appearance. Psychological Science.

[CR3] Christensen C, Heckerling PS, Mackesy ME, Berstein LM, Elstein AS (1991). Framing bias among expert and novice physicians. Academic Medicine.

[CR4] Christensen-Szalanski JJ, Brehmer B, Jungermann H, Lourens P, Sevon EG (1986). Improving the practical utility of judgment research. New directions in research on decision making.

[CR5] Croskerry P (2002). Achieving quality in clinical decision making: Cognitive strategies and detection of bias. Academic Emergency Medicine.

[CR6] Custers EJFM (2015). Thirty years of illness scripts: Theoretical origins and practical applications. Medical Teacher.

[CR7] Custers EJFM, Stuyt PMJ, De Vries Robbé PF (2000). Clinical problem analysis: A systematic approach to teaching complex medical problem solving. Academic Medicine.

[CR8] Cutrer WB, Sullivan WM, Fleming AE (2013). Educational strategies for improving clinical reasoning. Current Problems in Pediatric and Adolescent Health Care.

[CR9] Dawes RM, Faust D, Meehl PE (1989). Clinical versus actuarial judgment. Science.

[CR10] Dijksterhuis A (2004). Think different: The merits of unconscious thought in preference development and decision making. Journal of Personality and Social Psychology.

[CR11] Eddy DM, Kahneman D, Slovic P, Tversky A (1982). Probabilistic reasoning in clinical medicine: Problems and opportunities. Judgment under uncertainty: Heuristics and biases.

[CR12] Elstein AS, Shulman LS, Sprafka SA (1978). Medical problem solving. An analysis of clinical reasoning.

[CR13] Engel HPJ (2008). Tacit knowledge and visual expertise in medical diagnostic reasoning: Implications for medical education. Medical Teacher.

[CR14] Englebretsen G (2005). Bare facts and naked truths: A new correspondence theory of truth.

[CR15] Eva K (2005). What every teacher needs to know about clinical reasoning. Medical Education.

[CR16] Evans JSBT, Barston JL, Pollard P (1983). On the conflict between logic and belief in syllogistic reasoning. Memory & Cognition.

[CR17] Feltovich P, Spiro R, Coulson RL, Evans DA, Patel VL (1989). The nature of conceptual understanding in biomedicine: The deep structure of complex ideas and the development of misconceptions. Cognitive science in medicine. Biomedical modeling.

[CR18] Gigerenzer G, Brighton H (2009). Homo heuristicus: Why biased minds make better inferences. Topics in Cognitive Science.

[CR19] Gigerenzer G, Todd PM, ABC Research Group (1999). Simple heuristics that make us smart.

[CR20] Goldberg LR (1970). Man versus model of man: A rationale, plus some evidence, for a method of improving on clinical inference. Psychological Bulletin.

[CR21] Goldman GM (1990). The tacit dimension of clinical judgment. The Yale Journal of Biology and Medicine.

[CR22] Graber M, Gordon R, Franklin N (2002). Reducing diagnostic errors in medicine: What’s the goal?. Academic Medicine.

[CR23] Graber ML, Franklin N, Gordon R (2005). Diagnostic error in internal medicine. Archives of Internal Medicine.

[CR24] Hammond KR (1996). How convergence of research paradigms can improve research on diagnostic judgment. Medical Decision Making.

[CR25] Hammond KR (1996). Human judgment and social policy: Irreducible uncertainty, inevitable error, unavoidable injustice.

[CR26] Hammond KR (2000). Judgments under stress.

[CR27] Hammond KR (2007). Beyond rationality. The search for wisdom in a troubled time.

[CR28] Hobus PPM, Schmidt HG, Boshuizen HPA, Patel VL (1987). Contextual factors in the activation of first diagnostic hypotheses: Expert-novice differences. Medical Education.

[CR29] Johnson PE, Grazioli S, Jamal K, Zualkernan IA (1992). Success and failure in expert reasoning. Organizational Behavior and Human Decision Processes.

[CR30] Kahneman D (2011). Thinking, fast and slow.

[CR31] Kahneman D, Tversky A (1972). Subjective probability: A judgment of representativeness. Cognitive Psychology.

[CR32] Kanter SL, Brosenitsch TA, Mahoney JF, Staszewski J (2010). Defining the correctness of a diagnosis: Differential judgments and expert knowledge. Advances in Health Sciences Education.

[CR33] Kaufman DR, Patel VL (1991). Cognitive problem solving in the clinical interview: A cognitive analysis of the performance of physicians, residents, and students. Teaching and Learning in Medicine.

[CR34] Kirwan JR, Barnes CG, Davies PG, Currey HLF (1988). Analysis of clinical judgment helps to improve agreement in the assessment of rheumatoid arthritis. Annals of Rheumatic Diseases.

[CR35] Kuhn Th (1962). The structure of scientific revolutions.

[CR36] LeBlanc VR, Brooks LR, Norman GR (2002). Believing is seeing: The influence of a diagnostic hypothesis on the interpretation of clinical features. Academic Medicine.

[CR37] Lopes LL (1991). The rhetoric of irrationality. Theory & Psychology.

[CR38] Mamede S, Van Gog T, Van Den Berge K, Rikers RMJP, Van Saase JLCM, Van Guldener C, Schmidt HG (2010). Effect of availability bias and reflective reasoning on diagnostic accuracy among internal medicine residents. JAMA.

[CR39] McGuire CH (1985). Medical problem solving: A critique of the literature. Journal of Medical Education.

[CR40] Meijs, W. (2006). *Probabilistic measures of coherence*. Rotterdam, Netherlands: Erasmus University, Academic Thesis.

[CR41] Miller RA, Pople HE, Myers JD (1982). INTERNIST-I, an experimental computer-based diagnostic consultant for general internal medicine. The New England Journal of Medicine.

[CR42] Mosier KL (2009). Searching for coherence in a correspondence world. Judgment and Decision Making.

[CR43] Norman GR (2009). Dual processing and diagnostic errors. Advances in Health Sciences Education.

[CR44] Norman GR, Rosenthal D, Brooks LR, Muzzin LJ (1989). The development of expertise in dermatology. Archives of Dermatology.

[CR45] Patel VL, Groen GJ, Patel YC (1997). Cognitive aspects of clinical performance during patient workup: The role of medical expertise. Advances in Health Sciences Education.

[CR46] Patel VL, Kaufman DR, Higgs J, Jones M (1995). Clinical reasoning and biomedical knowledge: Implications for teaching. Clinical reasoning in the health professions.

[CR47] Peterson CR, Hammond KR, Summers DA (1965). Optimal responding in multiple-cue probability learning. Journal of Experimental Psychology.

[CR48] Polanyi M, Polanyi M (1969). The logic of tacit inference. Knowing and being.

[CR49] Pretz JE, Brookings JB, Carlson LA, Humbert TK, Roy M, Jones M, Memmert D (2014). Development and validation of a new measure of intuition: The types of intuition scale. Journal of Behavioral Decision Making.

[CR50] Prior AN, Edwards P (1967). Correspondence theory of truth. Encyclopedia of philosophy.

[CR51] Redelmeier D (2005). The cognitive psychology of missed diagnoses. Annals of Internal Medicine.

[CR52] Rescher N (2002). The coherence theory of truth.

[CR53] Sanders L (2009). Every patient tells a story. Medical mysteries and the art of diagnosis.

[CR54] Schiff, G. D., Seijeoung, K., Richard Abrams, R., Cosby, K., Lambert, B., Elstein, A. S., et al. (2005). Diagnosing diagnosis errors: Lessons from a multi-institutional collaborative project. In *Agency for healthcare research and quality, advances in patient safety: From research to implementation.* Washington, DC: DHHS. 21249820

[CR55] Shaffer VA, Hulsey L (2009). Are patient decision aids effective? Insight from revisiting the debate between correspondence and coherence theories of judgment. Judgment and Decision Making.

[CR56] Shortliffe E, Buchanan B (1975). A model of inexact reasoning in medicine. Mathematical Biosciences.

[CR57] Simon HA (1987). Making management decisions: The role of intuition and emotion. Academy of Management Executive.

[CR58] Spiro RJ, Feltovich PJ, Coulson RL, Anderson DK, Vosniadou S, Ortony A (1989). Multiple analogies for complex concepts: Antidotes for analogy-induced misconception in advanced knowledge acquisition. Similarity and analogical reasoning.

[CR59] Tape TG (2009). Coherence and correspondence in medicine. Judgment and Decision Making.

[CR60] Tape TG, Kripal J, Wigton RS (1992). Comparing methods of learning clinical prediction from case simulations. Medical Decision Making.

[CR61] Tape TG, Steele D, Wigton RS (1995). Learning to differentiate bacterial from viral meningitis: A non-linear judgment task with case simulations and feedback. Medical Decision Making.

[CR62] Thagard P, Verbeurgt K (1998). Coherence as constraint satisfaction. Cognitive Science.

[CR63] Tversky A, Kahneman D (1973). Availability: A heuristic for judging frequency and probability. Cognitive Psychology.

[CR64] Vicente KJ (1990). Coherence- and correspondence driven work domains: Implications for system design. Behaviour & Information Technology.

[CR65] White AR, Edwards P (1967). Coherence theory of truth. Encyclopedia of philosophy.

[CR66] Wigton RS (1988). Use of linear models to analyze physicians’ decisions. Medical Decision Making.

[CR67] Wigton RS (1996). Social judgement theory and medical judgement. Thinking & Reasoning.

[CR68] Woods NN, Brooks LR, Norman GR (2007). It all makes sense: Biomedical knowledge, causal connections and memory in the novice diagnostician. Advances in Health Sciences Education.

[CR69] Woods NN, Brooks LR, Norman GR (2007). The role of biomedical knowledge in diagnosis of difficult clinical cases. Advances in Health Sciences Education.

